# Teaching geriatric medicine through gamification: a tool for enhancing postgraduate education in geriatric medicine

**DOI:** 10.1007/s40520-021-01933-9

**Published:** 2021-07-18

**Authors:** Mathias Schlögl, Regina Elisabeth Roller-Wirnsberger, Susanne Sørensen Hernes, Stany Perkisas, Marit Stordal Bakken, Stéphanie Miot, Cafer Balci, Melanie Dani, Hanna Pajulammi, Paolo Piaggi, Clara Drenth-van Maanen, Katrin Singler

**Affiliations:** 1 Centre on Aging and Mobility, University Hospital Zurich and City Hospital Waid Zurich, Zurich, Switzerland; 2 Department of Internal Medicine, Medical University of Graz, Graz, Austria; 3 Department of Geriatric and Internal Medicine, Sorlandet Hospital Arendal, Sykehusveien 1, 4809 Arendal, Norway; 4 University Center for Geriatrics, University Hospital of Antwerp, Edegem, Belgium; 5 Department of Internal Medicine, Haraldsplass Deaconess Hospital, Bergen, Norway; 6 Department of Geriatrics, Montpellier University Hospital, Montpellier University, Montpellier, France; 7 Division of Geriatric Medicine, Eskişehir City Hospital, Eskisehir, Turkey; 8 Cutrale Perioperative and Ageing Group, Uren Biomedical Engineering Research Hub, Imperial College London, London, W12 0BZ UK; 9 Department of Geriatric Medicine, Central Hospital of Central Finland, Central Finland Health Care District, Jyväskylä , Finland; 10 Department of Information Engineering, University of Pisa, Pisa, Italy; 11 Department of Geriatric Medicine, University Medical Center Utrecht, Heidelberglaan 100, 3584 Utrecht, The Netherlands; 12 Department of Geriatric Medicine, Klinikum Nürnberg, Paracelsus Private Medical University, Prof. Ernst-Nathan-Str. 1, 90419 Nuremberg, Germany; 13 University Clinic for Acute Geriatric Care, City Hospital Waid Zurich, Zurich, Switzerland; 14 Department of Clinical Sciences, University of Bergen, Bergen, Norway; 15 CESP, INSERM U1178, Centre de recherche en Epidemiologie et Santé des Populations, Paris, France; 16 Expertise Centre Pharmacotherapy in Old Persons (Ephor), http://www.ephor.nl/en; 17 Institute for Biomedicine of Ageing, Friedrich-Alexander University Erlangen-Nürnberg, Kobergerstr. 60, 90408 Nürnberg, Germany

**Keywords:** Polypharmacy, Education, Training, Medical, Gamification, M&M game

## Abstract

**Background:**

Polypharmacy is becoming increasingly common and all doctors must be prepared to manage it competently.

**Aims:**

The aim of this project is to evaluate the feasibility and use of a novel gamification-based teaching intervention on polypharmacy among doctors undergoing advanced geriatric training. Among others, one of the learning goals for the students was to be able to describe the adherence to medication.

**Methods:**

Electronic questionnaire sent to students of the third session “evidence-based medicine in geriatrics” of advanced postgraduate course in geriatrics of the European Academy for Medicine of Ageing.

**Results:**

Most students reported issues with forgetting doses and remembering sufficiently to establish a medication routine due to busy schedules as well as social influences around medication taking. Reflecting on the challenges of the game, most students reported that their own prescribing practice was likely to change.

**Discussion and conclusion:**

The current model of learning appears to be a feasible approach for postgraduate medical education or in other areas of healthcare such as nursing or physiotherapy. Learning through action and reflection promotes deeper thinking and can lead to behavioral change, in this case thus enhancing the attitudes and understanding regarding pharmacological issues associated with ageing. Recommendations for future research in medical education about medication adherence are outlined.

## Background

Sustainable academic and clinical training is crucial to equip healthcare professionals with the knowledge, attitudes, and skills necessary for providing high quality care to a growing global population of older adults [1–3]. In the recent decade, experts in geriatric medicine and originating from all European Union (EU) Member States have launched training recommendations for Geriatric Medicine core competences on undergraduate level [4] and dedicated for training of all medical doctors across Europe as well as for specialist education using modified Delphi techniques [5]. Essential training requirements independent from level of education are knowledge, skills, and attitudes for delivery of tailored pharmacological care for ageing citizens [5]. Key element for this part of complex care management of older patients is building trust and relation with older clients to achieve and sustain adherence to medication regimens when treating older patients with polypharmacy [6].

There has been an extended academic debate around the educational tools and interventions to create an appropriate teaching, learning, and assessment strategy to ensure healthcare staff equipped for this demand [7]. In general, different theories of learning can be successfully applied to the teaching of geriatric medicine: experiential learning, reflection, motivation, and andragogy [8]. Several innovations have been shown to improve outcomes using technology to ensure the most effective allocation of teaching time and resources, using interprofessional education to improve attitudes towards older patients, and trying to engage patients in teaching [3]. One recently established approach to educational intervention focusing on higher levels of Bloom’s taxonomy [9] is the use “educational games”. These represent a type of experiential learning set, in which the learner “engages in some activity, looks back at the activity critically, abstracts some useful insight from the analysis and puts the results to work” [10, 11]. A variety of games have been used in medical education including ‘‘war games’’ to enhance high-risk clinical decision making [12], a quiz-type board game to teach medical microbiology [13], a ‘‘Survivor’’ game to review pulmonary physiology [14]. In 2010 Shiroma and colleges presented a “quiz-based” game to teach undergraduate medical students psycho-pharmacotherapy during a 6 week psychiatry clerkship for third year graduates [15]. Another approach is simulation training. Simulation is defined as a method “to replace or amplify real experiences with guided experiences, often immersive in nature, that evoke or replicate substantial aspects of the real world in a fully interactive fashion” [16]. Simulation training affords situational learning without compromising patient safety and focusses on trainees’ ability to display effective non-technical skills, crucial in the multidisciplinary environment, as well as their ability to manage clinical problems [17]. In this way, it provides a safe and effective learning platform that is also supported by adult educational theory [18, 19]. So far, there is only little to missing experience and evidence on gamification approach to train health care professionals during continuous professional education on topic of polypharmacy taking advantage of this novel educational approach.

Thus, the aim of the current study was to assess the acceptance and use of a novel gamification-based teaching intervention on polypharmacy among doctors undergoing advanced training in geriatrics during training sessions in the European Academy of Medicine of Ageing (EAMA) [20].

## Methods

The study took place during the third session “evidence-based medicine in geriatrics” of the XIIIth advanced postgraduate course in geriatrics of the EAMA from January 20 to 24, 2020 in Nice, France. Learning objectives of the training game were aligned with the preexisting objectives defined earlier [20] and outlined to students in advance. As the study only involved professionals and it referred to an educational method with EAMA students as participants, it did not raise any of the ethical issues flagged by the European Commission in the Horizon 2020 Programme Guidance “how to complete your ethics self-assessment”[21]. Therefore, ethics approval by an ethics review board/committee was deemed not to be necessary. Informed consent was asked and data were obtained anonymously.

### Learning goals for the polypharmacy training game

Students should know about and understand the principles of treatment including the effective and safe use of medicines as a basis for prescribing. Students should be able to describe the following concepts:

- Adherence to medication and factors affecting adherence in older people.
- The practice of safe and adequate prescribing in older people, taking account of differing physiology, drug interactions and multiple pathologies, and adverse drug reactions.
- Detection and management of drug underuse, overuse (including inappropriate medication use), and polypharmacy in older people.
- Integration of patient preferences and values into decisions about drug therapy.

### Equipment

Each participant received a small plastic bag containing multi-colored button-shaped chocolates and a corresponding medication plan, simulating a patient prescription (Table 1).

**Table 1:** The applied medication plan

Medication	Dose	Diagnosis	Dose regimen	Comment
Levothyroxine (25 µg orange, 50 µg brown)	75 µg	Hypothyroidism	1-0-0	30 min before the rest of the medication
Furosemide (blue)	40 mg	Arterial hypertension, ankle edema	1-1-0	
Ramipril (brown)	5 mg	Arterial hypertension	1-0-1	
Aspirin (yellow)	100 mg	Coronary heart disease	0-1-0	
Ibuprofen (Green)	600 mg	Knee pain, activated osteoarthritis	2×/day	Gap minimal 6 h, gap between Aspirin and Ibuprofen minimal 2 h
Alendronic acid (yellow)	70 mg	Osteoporosis	1×/week (Wednesday)	After intake of the drug stay in an upright position for minimal 30 min
Cholecalciferol (red)	1000 I.E	Osteoporosis	1-0-0	
Pantoprazole (orange)	20 mg	Stomach protection	0-0-0-1	As long as ibuprofen is on the medication plan

### Theoretical framework

EAMA students were asked to follow the prescription regimen during the one-week presence at the course venue. The participants were supposed to drive their learning forward by their own reflection, and the collective knowledge within the group.

**Fig. 1:**
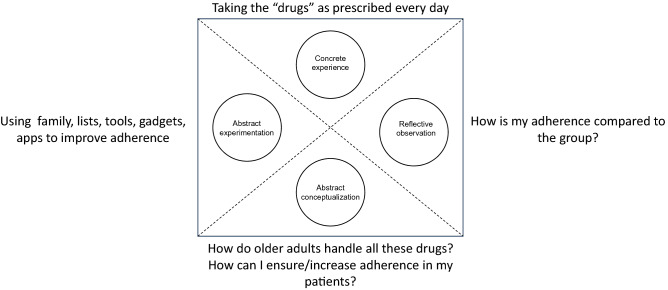
Theoretical framework. Based on the experiential design of the game follows Kolb’s original learning cycle [22], the teacher acts as a facilitator within the constructive framework, utilizing some connective elements. The participants were supposed to drive their learning forward by their own reflection, and the collective knowledge within the group.

The coordinating teacher (KS) acted as a facilitator within the constructive framework, utilizing some connective elements (Fig. 1).

### Practical approach

At baseline, we collected data on age and sex. For this, we assigned each student with an alphanumeric identification code, known only to that individual. After day 1, after day 2, and after day 3 we asked the students the following questions:

1. Did you take all your medications as prescribed yesterday (yes/no)?
2. How many medications did you not take?
3. If so, why?
4. Guess the adherence percentage of the group (0–100%)

After day 4, we additionally asked:

1. Did you take all your medications as prescribed yesterday (yes/no)?
2. How many medications did you not take?
3. Did you use any aids to help with drug adherence? If so, what?
4. What did you presume as the biggest obstacle to take your medication correctly?
5. What did you presume as most helpful to take your medication correctly?
6. Which medication did you forget to take most frequent?
7. Which medication did you forget to take least often?
8. If the chocolate candies had been real drugs, do you think your adherence would have improved?
9. Will the game affect your prescription practice? If so, how?
10. Reflecting the game during the last 4 days, can you name two learning goals that where most important for you?

### Analysis

We summarized the findings from question three on day 1–3 (“why did you not take the medication as prescribed?”) based on seven practical factors that can lead to unintentional non-adherence as previously shown in a recent published systematic review [23] (Table 2). To better analyze the association between group adherence prediction and medication adherence skills of participants, we performed an additional sensitivity analysis and created a group adherence prediction ratio (GAPR) defined as the relative variation of the group adherence prediction by the participant compared to the real group adherence (RGA). This real group adherence was defined for each day [1, 3] by the following formula, RGA = 100 – (sum of percentages of forgotten medication/total number of participant). The GAPR was deducted for each participant using the following formula, GAPR = – (RGA – participant group adherence estimation)/RGA and ranged from – 1 to + 1. A GAPR of – 1 corresponds to a 100% underestimation and a + 1 GAPR to a 100% of overestimation. Associations between this GAPR and the medication take (yes/no) on one side and the number of forgotten medications on the other side were estimated for each day using, respectively, ANOVA tests and linear regressions.

**Table 2:** Examples of the practical factors to medication adherence which contain practical barriers and their included factors

Theme number	Practical barrier category	Included factors
1	Formulation	–Taste, shape or size of tablets –Shape of tablets –Size of tablets –Swallowing difficulties –Inconvenience caused by injections (e.g., pain, bleeding, scars)
2	Instructions for use	–Dosing frequency –Total number of medicines needed to take –Storage of medication –Medication during travel or outside work (transport/storage) –Restrictions whilst on the medicine (e.g., on food/diet/alcohol/driving) –Administration requirements (at time of administration) –Variable dose pattern –Side effect burden
3	Issues with remembering	–Busy schedule (e.g., time needed to take medication) –Difficulties establishing medication routine
4	Capability Knowledge and skills	–Reading and understanding dispensing labels –Difficulties with opening container/packaging –Not understanding health provider instructions –Calculating correct dose –Cutting pills to get correct dose
5	Financial	–Direct: cost of medication –Indirect: travel fares, monitoring costs to treat your disease/other costs –General financial difficulties: meeting insurance or medication funding criteria
6	Medication supply	–Pharmacy does not have supply –Patient has run out of medications –Needing to obtain refills or scripts –Not having medicine on hand –Not knowing where or how to get supply
7	Social environment	–Social influences impeding medication taking –Embarrassment around medication taking –Stigma associated with certain medication

All statistics were performed using XLSTAT—life sciences, Adinsoft, France, 2020.

We summarized the findings from question ten (“reflecting the game during the last 4 days, can you name two learning goals that where most important for you?”) based on four themes which emerged from a recent prospective cohort study assessing resident’s knowledge of polypharmacy in The Initiative to Minimize Pharmaceutical Risk in Older Veterans (IMPROVE) [24]:

– Recognition of trade-offs in medication prescribing
– Acquisition of knowledge and skills
– Change of practice
– Value of interprofessional training

The Wilcoxon rank-sum test was used to assess changes in quantitative variables with skewed distribution (i.e., test scores) while the *χ*^2^ test was used for categorical variables.

## Results

The online survey was submitted to 47 students. On the first day, 33 students participated, 27 the second day, and 24 the third day. Of the 33 students (mean age: 39.6 years), 11 students (33%) took their medications after day 1 (*p* = 0.18) with no differences between sexes. The number of students taking all their medication remained stable although the number of students participating dropped to 25 after day 2 (*p* = 0.68), respectively, 24 after day 3 (*p* = 0.77). Practical barriers to medication adherence (question 3 on day 1–3, question 4 on day 4) are described below and representative quotations are shown in Table 3.

**Table 3:** The representative quotation of practical barriers to medication adherence

Practical barriers	Representative quotation
Issues with remembering –Day 1: *n* = 8 (24%) –Day 2: *n* = 11 (33%) –Day 3: *n* = 16 (49%)	• “I was late after the breakfast and forgot to go back in my room and totally forgot it at midday” • “I forgot one ibuprofen due to the lack of pain”
Social environment –Day 1: *n* = 4 (16%) –Day 2: *n* = 3 (12%) –Day 3: *n* = 3 (12%)	• “I did not take the medication at lunch and dinner because we were outside and I did not anticipated it” • “Again too busy day to remember the medications”
Instruction for use –Day 1: *n* = 8 (24%) –Day 2: *n* = 11 (33%) –Day 3: *n* = 16 (49%)	• “I think the timetable was not clear enough” • “I didn't understand why I have to take the medicine. I didn't have pain”
Capability—knowledge and skills –Day 1: *n* = 0 (0%) –Day 2: *n* = 2 (8%) –Day 3: *n* = 0 (0%)	• “I did not think I have to take the PPI”
Medication supply –Day 1: *n* = 0 (0%) –Day 2: *n* = 2 (8%) –Day 3: *n* = 0 (0%)	• “I forgot them in the room in the morning and did not have the time to get them afterwards”

### Issues with remembering

Most measures fell into this scheme. The students (day 1: *n* = 8 (24%); day 2: *n* = 11 (33%); day 3: *n* = 16 (49%) reported issues with remembering or forgetting doses or establishing a medication routine such as forgetting to take medication due to busy schedules.

### Social environment

Some students [day 1: *n* = 4 (16%); day 2: *n* = 3 (12%); day 3: *n* = 3 (12%)] reported problems related to social influences around medication taking.

### Instruction for use

A few of the students [day 1: *n* = 2 (8%); day 2: *n* = 1 (4%); day 3: *n* = 4 (16%)] identified items relating to issues with taking the medication as prescribed, such as dosing frequency, medication storage requirements or specific restrictions with medication administration such as the need to take with food or at certain times of the day.

### Capability—knowledge and skills

Only two students (day 1: *n* = 0; day 2: *n* = 2 (8%); day 3: *n* = 0) reported issues in understanding or following the specified instructions.

### Medication supply

This theme describes adherence barriers, which relate to obtaining or accessing medication supplies. It includes issues around the availability of medication and ease of supply of the medication. Only two students (day 1: *n* = 0; day 2: *n* = 2 (8%); day 3: *n* = 0) reported problems with this aspect.

### Formulation

The formulation theme related to factors around the specific medication formulation, such as the size of the oral dosage form (e.g., large tablets), which were identified to influence adherence. No students reported problems with this aspect.

### Financial

No students reported problems relating to finance or cost (e.g., direct costs, such as the cost of the medication, or indirect costs such as travelling expenses to obtain the medication).

### Answers—day 4

On day 4, 24 out of the 33 students took all their medications (73%). Some of the students (*n* = 10; 42%) used different aids to help with drug adherence (tailoring/routinisation: *n* = 6 (25%) (e.g., putting the instructions and medications in a place where the participant would see them when entering the room); use of adherence aids (e.g., using a reminder on the cell phone): *n* = 3 (13%); pillbox: *n* = 1 (4%). The biggest obstacle to take the medication correctly was instruction for use (*n* = 11; 46%), social environment (*n* = 5; 21%), capability (*n* = 3; 13%) and issues with remembering (*n* = 1; 4%). According to the students, tailoring (*n* = 12; 50%), the use of adherence aids (*n* = 4; 17%), the use of a pillbox (*n* = 4; 17%), and a medication list (*n* = 3; 13%) were presumed as most helpful to take the medication correctly. Interestingly, the medication the students forgot the most frequent were Ibuprofen (*n* = 8; 33%), Pantoprazole (*n* = 2; 8%), and Furosemide (*n* = 1; 4%). There was no clear signal which specific medication the students forgot the least often, however, seven students reported that they did not forget the morning medication. Out of the 24 students, 58% (*n* = 14) reported that their adherence would have been improved if the medication had been real, whereas 29% (*n* = 7) answered otherwise and 13% (*n* = 3) answered maybe. Interestingly, the majority of the students (*n* = 20; 83%) answered that the game would affect their prescription practice. In detail, the majority (*n* = 14; 70%) answered that it would affect their own understanding and knowledge whereas (*n* = 6; 30%) answered that it would affect their own behavior. Finally, reflecting upon the two learning goals that were most important to the students, the majority (day 1: *n* = 19; 79%) answered that they recognized the trade-offs in medication prescribing and a potential change in their own prescribing practice (day 2: *n* = 9; 38%).

### Assessment of the ratio of prediction of group adherence by participant and its association with medication take and forgotten medication

For day 1, medication take was not significantly associated with GAPR (*p* value 0.114), but the high number of forgotten medications was significantly associated with a negative GAPR (*p* value 0.32). For days 2 and 3 we observed a significant association between medications take and high GAPR (*p* value, respectively, 0,029 and 0,048) and the number of forgotten medications was significantly associated with a low GPAR (*p* value, respectively, 0,01 and 0,012). The participants who missed more treatments underestimated the medication adherence of the group more frequently. The more the participant is able to take his medication, the more he thinks that the group adherence is good.

## Discussion

The aim of the current study was to assess the feasibility and use of a novel gamification-based teaching intervention on polypharmacy among doctors in advanced training in geriatrics. Medication management is an important component of medical education, particularly in geriatric medicine. The Association of American Medical Colleges has put forth 26 minimum geriatrics competencies under eight domains for graduating medical students; medication management is one such domain [25].

Overall, the current approach seems both feasible and applicable as a new interactive-learning method in postgraduate geriatric medicine education.

Importantly, there is a lack of uniformity in the definitions of the main forms of game-based learning—gamification, serious games, and simulations [19, 26]. As stated in a recent systematic review by van Gaalen and colleagues on empirical evidence for the effectiveness of gamification approaches, gamification refers to using game attributes in a non-gaming context [26]. Simulation can be defined as a situation in which a particular set of conditions is created artificially to study or experience something that could exist in reality. Simulations provide instant feedback on performance, which is delivered as accurate and realistic as possible in a safe environment. In contrast to gamification, simulations do not need game elements like a scoring system and a win/lose condition. However, game-design techniques and solutions can be employed to create the simulated reality and the experience of something real. Simulations are, therefore, best seen as learning activities that necessarily carry some game intention, but do not use game elements [26].

Based on our current approach [23, 24], we were able to identify and synthesize valuable practical factors to medication adherence. Based on our sensitivity analysis we could also show that the participants who missed more treatments underestimated more frequently the medication adherence of the group. Therefore, a geriatrician with own good skills for treatment adherence could overestimate the medication adherence of his or her patients and/or could consider less issues in medication adherence of his patients. Nevertheless, we can observe a negative group adherence prediction ratio, indicating that in general participants underestimated the group adherence. Future learning goals should also highlight those cognitive biases of participants to improve their approach of polymedication.

In 2011, Gellad and colleagues performed systematic review describing potential nonfinancial barriers to medication adherence among seniors [27]. Some potential barriers (i.e., factors associated with non-adherence) were identified from the nine included studies, including patient-related factors such as disease-related knowledge, health literacy, and cognitive function; drug-related factors such as adverse effects and polypharmacy; and other factors including the patient-provider relationship and various logistical barriers to obtaining medications [27]. In the current study, most students reported issues with remembering, forgetting doses or establishing a medication routine due to busy schedules as well as social influences interrupting medication taking. Based on a recent review assessing the reasons for non-adherence, frequent medication review, and knowledge regarding the purpose of the medication were positively associated with adherence [28]. Factors associated with poor adherence were multimorbidity, cognitive impairment, complex regimens with multiple prescribing physicians, and problems with drug storage or formulation. The findings of this narrative review suggest that interventions to improve adherence could focus on medication review, simplifying regimens, and educating patients about their treatment [28].

In 2017, Marcum and colleagues provided an updated evidence summary from randomized controlled studies to determine whether interventions aimed at improving medication adherence also improve the health outcomes of older adults residing in community-based settings [29]. Across the 12 included studies, interventions were grouped into three main categories: behavioral/educational (*n* = 3), pharmacist-led (*n* = 7), and reminder/simplification (*n* = 2). Among the behavioral/educational intervention studies, two showed improvements in both adherence and related health outcomes, whereas one found no changes in adherence or health outcomes. Among the pharmacist-led studies, three showed improvements in both adherence and related health outcomes, while three reported no changes in adherence or health outcomes. One found an improvement in adherence but not health outcomes. Among the reminder/simplification studies, both studies reported improvements in adherence without a significant impact on related health outcomes [29]. In the current study, 42% of the students also used different reminders/simplifications to help with drug adherence.

There are some limitations to this study. First, we did not perform a pre-/post-evaluation on the knowledge levels of the participants; thus, we have no real data on whether the educational event improved the knowledge of pharmacological issues associated with ageing. Second, the time reserved for the event was limited. When organizing similar events in the future, we would suggest at least 15 minutes to be reserved for each subdomain of the game to guarantee enough time for discussion. Third, although the results of this pilot are very promising, the number of participants is relatively low and the group of students comes from different countries. Low statistical power might reduce the chance of detecting a true effect [30]. Furthermore, it is possible that the estimate of the magnitude of that effect provided by the study is exaggerated. This effect inflation is often referred to as the "winner's curse" and is likely to occur whenever claims of discovery are based on thresholds of statistical significance (for example, *p* < 0.05) [31]. Fourth, the results might not be generalizable to other postgraduate settings in geriatric medicine due to the unique nature of the EAMA program. The EAMA was founded in 1992 as an “advanced postgraduate course in geriatric medicine”, to train future key opinion leaders in geriatric medicine. EAMA students are selected based on structured criteria of workforce experience and academic background, but may still differ in their level of expertise, individual constraints and preferences when starting the program [20]. In general, EAMA students are highly engaged and motivated students, introducing a possible selection bias. However, since the students volunteered to participate, there still may have been some bias in the engagement student’s engagement levels. Furthermore, the results might be subject to recall bias.

## Conclusion

In summary, the current training approach has been proven useful for continuous professional education and might be easily transferred to various educational settings, also less advanced ones and also in interprofessional education. Learning through action and reflection promotes deeper thinking and can lead to a transformation, in this case thus enhancing the attitudes and understanding regarding pharmacological issues associated with ageing.

Based on the findings of the study, future research [27, 29] should focus on:

- Standardizing medication adherence measurements among older adults to gain a better understanding of this important issue,
- Successful interventions involving behavioral/educational and pharmacist interventions,
- Developing and testing patient-centered and multidisciplinary interventions using evidence-based principles to improve medication adherence and health outcomes in older adults.

## Data Availability

The datasets used and analyzed during the current study are available from the corresponding author on reasonable request.

## References

[1] Science Advice for Policy by European Academies (2019) Transforming the future of ageing, Berlin. Report no: 978-3-9820301-1-1

[2] World Health Organization (2016) Health workforce for ageing populations. World Health Organization Department of Ageing and Life-Course

[3] Oakley R, Pattinson J, Goldberg S (2014). Equipping tomorrow’s doctors for the patients of today. Age Ageing.

[4] Masud T, Blundell A, Gordon AL (2014). European undergraduate curriculum in geriatric medicine developed using an international modified Delphi technique. Age Ageing.

[5] Roller-Wirnsberger R, Masud T, Vassallo M (2018). European postgraduate curriculum in geriatric medicine developed using an international modified Delphi technique. Age Ageing.

[6] Coombes I, Mitchell C, Stowasser D (2007). Safe medication practice tutorials: a practical approach to preparing prescribers. Clin Teach.

[7] Kennedy MB, Malik M, Haq I (2016). Safe prescribing training provision for junior doctors: is this optimal?. BMC Med Educ.

[8] Roller-Wirnsberger Regina SK, Marie Christina P (2018). Learning geriatric medicine: a study guide for medical students practical issues in geriatrics.

[9] Bloom BS, Engelhart MD, Furst EJ (1956). Taxonomy of educational objectives: the classification of educational goals.

[10] Nevin CR, Westfall AO, Rodriguez JM (2014). Gamification as a tool for enhancing graduate medical education. Postgrad Med J.

[11] Alfarah Z, Schünemann HJ, Akl EA (2010). Educational games in geriatric medicine education: a systematic review. BMC Geriatr.

[12] Hedrick TL, Young JS (2008). The use of “war games” to enhance high-risk clinical decision-making in students and residents. Am J Surg.

[13] Beylefeld AA, Struwig MC (2007). A gaming approach to learning medical microbiology: students’ experiences of flow. Med Teach.

[14] Howard MG, Collins HL, DiCarlo SE (2002). “Survivor” torches “who wants to be a physician?” in the educational games ratings war. Adv Physiol Educ.

[15] Shiroma PR, Massa AA, Alarcon RD (2011). Using game format to teach psychopharmacology to medical students. Med Teach.

[16] Ross AJ, Anderson JE, Kodate N (2013). Simulation training for improving the quality of care for older people: an independent evaluation of an innovative programme for inter-professional education. BMJ Qual Saf.

[17] Fisher JM, Walker RW (2014). A new age approach to an age old problem: using simulation to teach geriatric medicine to medical students. Age Ageing.

[18] Braude P, Reedy G, Dasgupta D (2015). Evaluation of a simulation training programme for geriatric medicine. Age Ageing.

[19] Plakiotis C (2017). Clinical simulation training in geriatric medicine: a review of the evidence and lessons for training in psychiatry of old age. Adv Exp Med Biol.

[20] Roller-Wirnsberger RE, van den Noortgate N, Bonin-Guillaume S (2018). Setting competencies and standards for a European leadership program in geriatric medicine: “the European Academy for Medicine of Ageing (EAMA) reloaded”. Euro Geriatric Med.

[21] European Commission (2019) Horizon 2020 Programme. Guidance. How to complete your ethics self-assessment. European Commission, Directorate-General for Research & Innovation. https://ec.europa.eu/research/participants/data/ref/h2020/grants_manual/hi/ethics/h2020_hi_ethics-self-assess_en.pdf

[22] Kolb DA (2015). Experiential learning: experience as the source of learning and development.

[23] Chan AHY, Cooper V, Lycett H, et al (2020) Practical barriers to medication adherence: what do current self- or observer-reported instruments assess? Front Pharmacol 11 10.3389/fphar.2020.00572 PMC7237632 32477110

[24] Mecca MC, Thomas JM, Niehoff KM (2019). Assessing an interprofessional polypharmacy and deprescribing educational intervention for primary care post-graduate trainees: a quantitative and qualitative evaluation. J Gen Intern Med.

[25] Ramaswamy R (2013). How to teach medication management: a review of novel educational materials in geriatrics. J Am Geriatr Soc.

[26] van Gaalen AEJ, Brouwer J, Schönrock-Adema J (2021). Gamification of health professions education: a systematic review. Adv Health Sci Educ Theory Pract.

[27] Gellad WF, Grenard JL, Marcum ZA (2011). A systematic review of barriers to medication adherence in the elderly: looking beyond cost and regimen complexity. Am J Geriatric Pharmacother.

[28] Smaje A, Weston-Clark M, Raj R (2018). Factors associated with medication adherence in older patients: a systematic review. Aging Med (Milton).

[29] Marcum ZA, Hanlon JT, Murray MD (2017). Improving medication adherence and health outcomes in older adults: an evidence-based review of randomized controlled trials. Drugs Aging.

[30] Button KS, Ioannidis JP, Mokrysz C (2013). Power failure: why small sample size undermines the reliability of neuroscience. Nat Rev Neurosci.

[31] Ioannidis JPA (2008). Why most discovered true associations are inflated. Epidemiology.

[32] Council of the European Communities (2000). Charter of fundamental rights of the European Union. Off J Eur Communities.

[33] European Court of Human Rights (1953) European convention on human rights. https://www.echr.coe.int/Documents/Convention_ENG.pdf

